# Genome-Wide Analysis and Evolution of the Pto-Like Protein Kinase (PLPK) Gene Family in Pepper

**DOI:** 10.1371/journal.pone.0161545

**Published:** 2016-08-18

**Authors:** Jelli Venkatesh, Molly Jahn, Byoung-Cheorl Kang

**Affiliations:** 1 Department of Plant Science and Plant Genomics and Breeding Institute, Vegetable Breeding Research Center, Seoul National University, Seoul, 151–921, Korea; 2 University of Wisconsin, Madison, Wisconsin, WI 53706, United States of America; Texas A&M University College Station, UNITED STATES

## Abstract

The tomato *Pto* gene, which encodes a serine/threonine kinase (STK) domain-containing protein, confers resistance to bacterial speck disease caused by *Pseudomonas syringae* pv. tomato (*Pst*). In this study, *in vivo* recognition assays using PVX constructs showed that AvrPto was specifically recognized in the pepper genotypes. This AvrPto recognition caused a nonhost hypersensitive response (HR) and localization of the PVX::AvrPto fusion protein to inoculated pepper leaf tissues, which indicates the presence of a similar Pto recognition mechanism in pepper as in tomato. However, genome-wide analysis in pepper revealed no Pto clade corresponding to that in tomato, suggesting an alternative system for Pto recognition in pepper. Nevertheless, 25 Pto-like protein kinases (PLPKs) with a highly conserved STK domain have been identified in the pepper genome. For the majority of the amino acid sites in the STK domain of Ptos and PLPKs, nonsynonymous (dN) to synonymous (dS) nucleotide substitution ratios (ω) were less than one, suggesting that purifying selection played a predominant role in the evolutionary process. However, some amino acid sites were found to be subjected to episodic positive selection in the course of evolution of Pto homologs, and, thus, different evolutionary processes might have shaped the Pto gene family in plants. Based on RNA-seq data, *PLPK* genes and other Pto pathway genes, such as *Prf*, *Pti1*, *Pti5*, and *Pti6* were expressed in all tested pepper genotypes. Therefore, the nonhost HR against *Pst* in pepper may be due to the recognition of the AvrPto effector by a PLPK homolog, and subsequent action of downstream components of the Pto signaling pathway. However, the possibility remains that the recognition of AvrPto in pepper plants may involve activities of other receptor like kinases (RLKs). The identification of the PLPKs in this study will serve as a foundation for further efforts to understand the roles of PLPKs in nonhost resistance.

## Introduction

Plants have developed various defense responses to pathogens. Disease resistance often occurs in a gene-for-gene manner through interaction between proteins encoded by the plant disease resistance (*R*) genes and the corresponding effector proteins encoded by pathogen avirulence (*Avr*) genes. Such interactions activate a cascade of defense-related responses in the host to suppress the pathogen attack [1, 2]. Several plant *R* genes have been cloned and progress has been made to understand the host resistance mechanisms [3–8].

Plant *R* genes are classified into eight classes based on the presence of conserved domains [7, 9]. The *Pto* gene in tomato (*Solanum pimpinellifolium* L.) is among the best characterized *R* genes [10]. *Pto* encodes a serine-threonine kinase (STK) and confers resistance to *P*. *syringae* strains that express effector protein AvrPto or AvrPtoB [11–13]. The Pto kinase interacts directly with AvrPto and AvrPtoB, thereby triggering immunity in the host plant [12, 14, 15]. Both AvrPto and AvrPtoB have been shown to enhance virulence when expressed in bacterial strains in which they do not occur naturally [16–18]. AvrPtoB functions in pathogenesis as an inhibitor of programmed cell death (PCD) disrupting host defense responses [18]. Interestingly, AvrPto is also capable of suppressing PCD induced by nonhost pathogens in *Nicotiana benthamiana* and tomato [19]. Three classes of downstream effectors have been identified in the *Pst* resistance system. Pti1 is a protein kinase that enhances the hypersensitive response *in vivo*; Pti4, Pti5, and Pti6 are defense-related EREBP-like transcription factors [16, 20, 21] and Prf is a nucleotide-binding leucine-rich repeat (NB-LRR) protein. The phosphorylation of the Pto kinase by AvrPto effector is sensed by Prf, inducing effector-triggered immunity [22–25].

Despite extensive studies of the Pto pathway genes in plants, particularly in tomato, no functional homologs of Pto that recognize AvrPto have been identified in any genus other than *Solanum*. There have been efforts to amplify Pto-like sequences using several plant species [26, 27]. In these studies, it was suggested that the AvrPto-recognizing molecule may be similar to Pto kinase from tomato. However, mutation analysis has shown that the AvrPto protein is differentially recognized by tomato and tobacco [17], suggesting that the AvrPto-recognizing protein in tobacco may be unrelated to the tomato *Pto* sequence [17]. Recognition of AvrPto has been reported in soybean [28], suggesting that AvrPto recognition and downstream signaling cascades may be conserved across plant species [28]. The recognition of AvrPto by these plant species also raises a question about the role of the AvrPto-recognizing proteins in nonhost species of *Pst*.

Recently, *Pto*-like genes have been identified in several plant species including some *Solanum* species [27], common bean [26], grapevine [29], cucumber [30], banana [31], strawberry [32], and *Citrus* [33]. Notably, *Pto* orthologs are not present in two different *S*. *lycopersicum* genotypes [34, 35], whereas the AvrPto-specific Pto-mediated resistance has likely been introgressed from *S*. *pimpinellifolium* in some cultivated *S*. *lycopersicum* varieties [10, 36]. Despite advances in the understanding of the molecular genetics of *Pto* genes, the molecular evolution of the *Pto* gene remains poorly understood. Understanding the evolution of the *Pto* genes is important to unravel their functional divergence as well as to understand their role in nonhost disease resistance.

Pepper, an important spice crop, is considered a nonhost of *Pst*. Several lines of evidence suggest that pepper may have the ability to recognize the AvrPto protein and induce resistance [19]. However, Pto kinase has not been established as the recognizing component. In this study, we tested whether pepper can recognize AvrPto using *in vivo* recognition assays with a PVX system. In addition, we performed genome-wide analysis of the Pto-like gene family (PLPKs) in pepper and identified 25 full-length *PLPK* genes, which were classified into eight subclasses (PLPK I to PLPK VIII) based on sequence similarity and phylogenetics. To gain insight into the evolutionary diversity of the plant Pto-like genes, comparative phylogenetic and molecular evolutionary analyses of the Pto gene family were performed, using *Pto* genes from other Solanaceae family crops, such as tomato, potato, and *N*. *benthamiana*, as well as *Arabidopsis* and rice. Structural characteristics of pepper PLPKs were investigated using various computational tools. Finally, expression profiles of the *PLPK* genes from various pepper genotypes were investigated using RNA-seq expression data.

## Materials and Methods

### Plant materials

*Capsicum* genotypes *C*. *annuum* ‘NuMex RNaky’ (RNaky), ‘Early CalWonder 30’ (ECW), both provided by Robert Stall, and ‘Criollo de Morelos 334’ (CM334), ‘Perennial’, *C*. *chinense* ‘PI159234’ (234) and ‘Habanero’ were used in this study. The experimental lines were either typical commercial varieties that are susceptible to all agriculturally significant pathogens (ECW and RNaky) or varieties with resistance to various pathogens or diseases (Perennial, CM334, Habanero, and PI159234). *N*. *benthamiana* was used for PVX multiplication.

### Transient expression of AvrPto in pepper using PVX-derived vectors

For transient expression assays using PVX-derived vectors, AvrPto and the mutant AvrPto^I96T^ were provided by G. Martin and X. Tang respectively, and cloned into pPVX201 provided by D. Baulcombe [37]. The 528-bp coding regions of each *AvrPto* gene was amplified by PCR from the plasmids pPtE6 [28] and pPtE6:I96T [17] using the primers 5'-ATATCGATGGGAAATATATGTGTC-3' and 5'-GAGGTCGACATTATGACGCC-3'. The introduced *Cla*I and *Sal*I sites in the primers are underlined. The amplified fragments were cloned into pGEM^T^ (Promega, Madison, WI) and confirmed by sequencing. The products were digested with *Cla*I and *Sal*I and subcloned into corresponding vector sites. The resulting pPVX201 derivatives were designated pPVX201::AvrPto and pPVX201::AvrPto^I96T^. A derivative of pPVX201 carrying GFP cDNA, pPVX204, was used as a control [37]. For inoculum production, 4–6 week old *N*. *benthamiana* plants were mechanically inoculated with ≥50 μg plasmid in a 15% bentonite suspension in 43 mM sodium phosphate buffer (pH 7.0) [37]. Systemically infected *N*. *benthamiana* plants inoculated with pPVX201, pPVX204 or pPVX::AvrPto^I96T^ developed systemic mosaic symptoms 14 days post-inoculation (dpi), whereas pPVX::AvrPto induced bleaching and mottling followed by systemic necrosis [38]. Non-necrotic symptomatic *N*. *benthamiana* leaves were homogenized in 50 mM phosphate buffer (pH 7.0) 10–14 dpi and used to mechanically inoculate 6–8 week old pepper seedlings. Mock-inoculated pepper controls were routinely included. Infection was confirmed by hybridization analysis and positive indirect enzyme-linked immunosorbent assay (ELISA) using [39] anti-PVX antibody (Agdia, Elkhart, IN). Total RNA was prepared from uninoculated upper leaves of inoculated plants 14 dpi, blotted onto Hybond N^+^, and hybridized with radiolabeled PVX-derived and *AvrPto* sequence.

### Genetic mapping and genome-wide analysis of Pto pathway homologs

The previously published *Capsicum* genetic linkage map [40] was used to determine map positions of Pto-pathway genes. *Pto*, *Fen*, and *Prf* cDNA clones were provided by S.D. Tansksley (Cornell University, Ithaca, NY). *Pti1*, *Pti4*, *Pti5*, and *Pti6* clones were provided by G. Martin (Cornell University, Ithaca, NY). To investigate RFLPs between PI159234 and NuMex RNaky, survey filters containing parent DNA digested with the restriction enzymes *Eco*RI, *Eco*RV, *Dra*I, *Bcl*I, *Bst*NI, *Hind*III, and *Xba*I were hybridized with the Pto-pathway genes. The F_2_ mapping of 75 plants described by Livingstone *et al*.[40] was used to collect RFLP segregation. The genetic map positions of the cloned PCR fragments were determined relative to the original framework marker data set with MAPMAKER software.

The Pto gene family members were identified in the pepper (*C*. *annuum*) genome through BLAST searches using the CM334 20140109 (v1.5) PROTEINS database (http://cab.pepper.snu.ac.kr/) with the *Pto* gene homolog sequences, *LpimPth2* (AAF76305), *LpimPth3* (AAF76304), *LpimPth4* (AAF76303), *Fen_kinase* (AAF76307), and *Pto_kinase* (AAF76306) from *S*. *pimpinellipolium* as queries. Predicted Pto protein homolog sequences from potato were obtained from the Solanaceae Genomics Resource database (http://solanaceae.plantbiology.msu.edu/). Pto-like protein kinase sequences from *Arabidopsis* and rice were obtained through BLAST searches at The *Arabidopsis* Information Resource database (TAIR) and Rice Genome Annotation Project (http://rice.plantbiology.msu.edu/index.shtml) using tomato Pto homolog sequences as queries. Pto-like protein sequences from tomato and tobacco were obtained through BLASTP searches using the Sol Genomics Network (https://solgenomics.net/) database and tomato Pto sequences as queries. We also identified homologs of tomato *Prf* and pepper *Pti* homologs through BLAST searches using tomato *Prf1* (AAF76308), *Pti1* (U28007), *Pti4* (U89255), *Pti5* (U89256), and *Pti6* (U89257) sequences as queries.

### Chromosomal location of the pepper Pto-like genes and other pepper genes related to Pto pathway

The chromosomal locations of the *PLPK* genes and other Pto pathway genes in pepper were obtained from the pepper genome database (http://cab.pepper.snu.ac.kr/). The *PLPK* genes were mapped to the pepper chromosomes using the MapChart program [41]. Genomic DNA and CDS sequences of the pepper genes were obtained from pepper genome database (http://cab.pepper.snu.ac.kr/). Exon-intron structure of the *PLPK* genes and other Pto pathway genes was visualized by comparing genomic DNA sequences with their corresponding CDS sequence with the gene structure display server (http://gsds.cbi.pku.edu.cn/).

### Phylogenetic analysis and identification of conserved motifs

To establish evolutionary relationships, protein sequences from various plant species including pepper, tomato, potato, *N*. *benthamiana*, *Arabidopsis*, and rice were included in the phylogenetic tree. To determine phylogenetic relationships between PLPKs in pepper and in other plant species, a multiple sequence alignment was generated including all available PLPK sequences from plant species such as rice, *Arabidopsis*, tomato, and *N*. *benthamiana*, with the predicted pepper PLPKs using the Clustal Omega program with default parameters (http://www.ebi.ac.uk/Tools/msa/clustalo/). Various classes of *Arabidopsis* protein kinases [42] were also included in the phylogenetic analyses to distinguish Pto and PLPK members from other classes of protein kinases. Aligned sequences with highly divergent regions or gaps resulting in uncertain alignments were edited using JalView 2.8 [43] and were excluded from further analysis. Phylogenetic analysis of full-length PLPK sequences was performed using the MEGA 6.0 software with the neighbor-joining (NJ) method [44], and the bootstrap analysis was carried out with 1000 iterations. Similarly, a separate phylogenetic analysis on the PLPKs from pepper was performed. The tree was rooted with pepper receptor like kinase (RLK) protein. The pepper PLPK members were classified based on their phylogenetic relationship with PLPK members from other plant species. Subcellular localization of the pepper PLPKs was predicted using the EuLoc web server [45]. The structural and functional domains of the PLPKs were analyzed using the SMART program (http://smart.embl-heidelberg.de/).

### Detection of selection pressure

To explore the nature of the selection pressure on the serine/threonine kinase (STK) domain of the Pto and PLPK residues, nonsynonymous (dN) and synonymous (dS) substitutions, and the dN/dS ratio (ω) for each nucleotide codon were estimated using the Datamonkey web server (http://www.datamonkey.org/). To avoid false positives, an automated genetic algorithm recombination detection (GARD) analysis was performed [46]. The site-specific selection pressure was calculated using several codon-based maximum-likelihood methods: fixed-effect likelihood (FEL), internal fixed-effect likelihood (IFEL), and single likelihood ancestor counting (SLAC), as implemented in the Datamonkey web interface [47]. Codon-based nucleotide sites were investigated using the REV nucleotide substitution model. To identify the individual codons that are subject to diversifying selection, the mixed effects model of evolution (MEME) analysis was performed. A ω value <1 indicates sites under negative (purifying) selection, a ω value >1 indicates sites under (diversifying) selection, and a ω value = 1 indicates sites that are not subject to selection pressure.

### RNA-seq expression data analysis

The expression patterns of *PLPK* genes from various pepper genotypes were analysed using RNA-seq data from previous research [48]. The sequence reads were mapped to the pepper transcriptome database (http://cab.pepper.snu.ac.kr) with CLC Genomics Workbench v8.0 (CLC Bio, Aarhus, Denmark) with default parameters. Expression values were measured in RPKM (reads per kilobase of exon model per million mapped reads). The RPKM values were log2-transformed and the heat map was generated using the heatmap.2 function from the ggplots in R-package.

## Results

### AvrPto specifically induces HR and resistance to PVX in pepper

**Fig 1:**
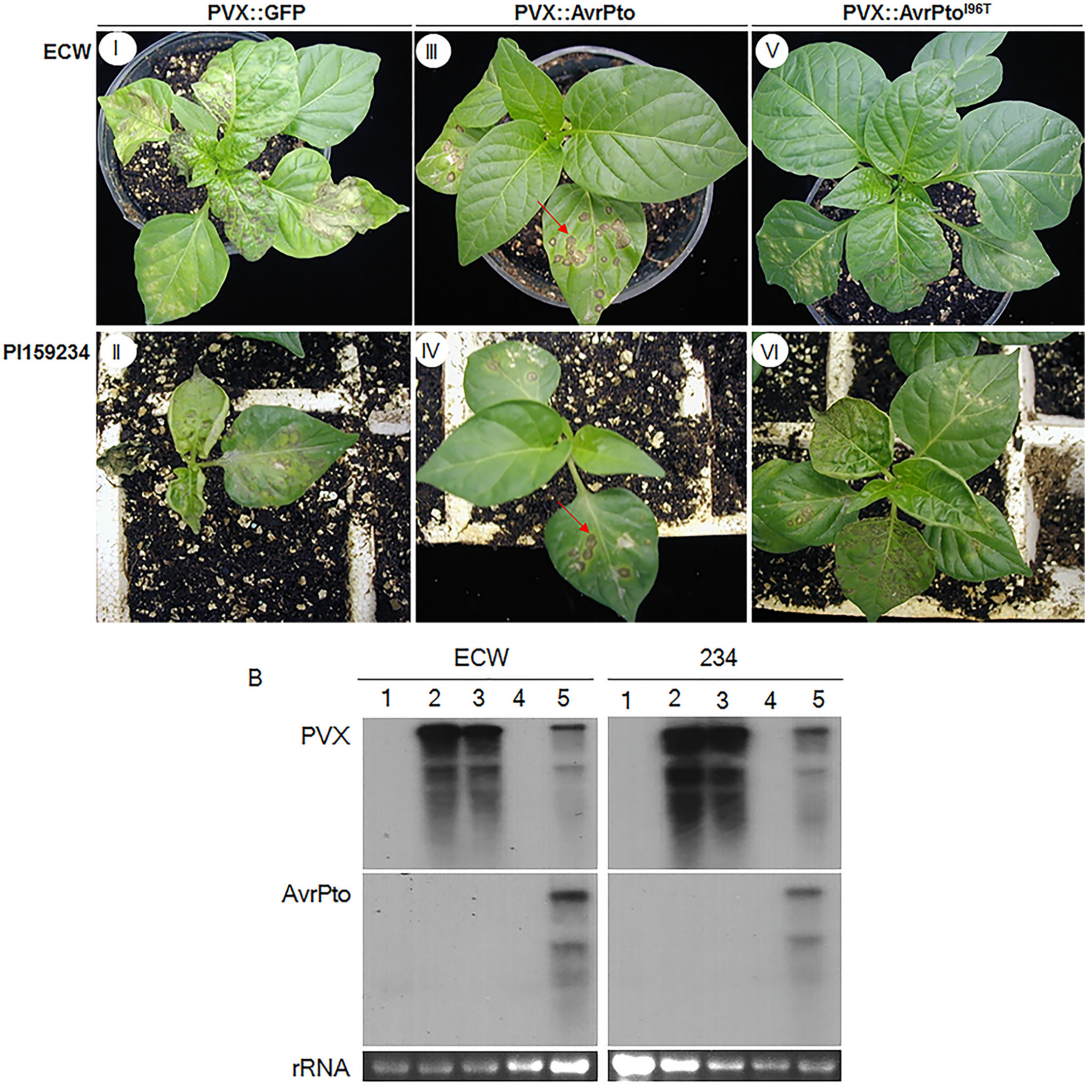
Pepper seedlings inoculated with PVX vectors for expression of GFP, AvrPto and AvrPto^I96T^. A: Transient expression of AvrPto and AvrPto^I96T^ using a PVX-derived vector system in *C*. *annuum* ‘ECW’ (upper panels) and *C*. *chinense* ‘PI159234’ (lower panels). Plants were photographed at 14 dpi. Panels I, II: pPVX::GPF; Panels III, IV: pPVX::AvrPto; Panels V, VI: pPVX::AvrPto^I96T^. B: Gel blot hybridization analysis of PVX RNA accumulation and the presence of *AvrPto* in uninoculated upper leaves of *C*. *annuum* ECW and *C*. *chinense* ‘PI159234’ infected with pPVX vector constructs. Total RNA was prepared from upper uninoculated leaves 14 dpi with the PVX constructs above or from mock-inoculated plants, blotted onto Hybond N^+^, and hybridized with a radiolabeled PVX-derived sequence (upper panels) and AvrPto (middle panels). Lane 1, mock-inoculated control; Lane 2, pPVX vector alone; Lane 3, pPVX::GFP; Lane 4, pPVX::AvrPto; and Lane 5, pPVX::AvrPto^I96T^. The lower panel shows ethidium bromide-stained ribosomal RNA prior to blotting.

To determine whether specific recognition of AvrPto occurs in pepper, PVX-derived vectors [49] were used to express AvrPto and AvrPto^I96T^, a mutant with a substitution at position 96 that prevents the interaction between AvrPto and Pto *in vitro* and *in planta* with no effect on virulence [17]. In a preliminary study, six different pepper genotypes, including Perennial, 234, Habanero, Rnaky, ECW, and CM334 were screened for susceptibility to PVX (S1 Fig). When inoculated with the vector alone, 234, Habanero, ECW, and RNaky genotypes developed viral symptoms throughout the plant (S1 Fig). 234 and ECW genotypes were subsequently grown and inoculated with the PVX vector alone or a construct expressing GFP, AvrPto, or AvrPto^I96T^ (Fig 1). Inoculation with the vector alone (S1 Fig) and *GFP*-containing PVX constructs (Fig 1A panels I, II) resulted in systemic symptoms and infection was confirmed by the presence of viral antigen and PVX-homologous sequences in both inoculated (data not shown) and upper uninoculated tissue (Fig 1), and in the case of *GFP*-containing constructs by fluorescence of uninoculated leaves under UV light (data not shown). By contrast, when plants were inoculated with pPVX::AvrPto, symptoms were restricted to lower inoculated leaves, which displayed localized necrotic lesions (Fig 1A panels III, IV). In addition, PVX antigen and PVX- and AvrPto-homologous sequences were detected only in inoculated tissue (data not shown), and not in uninoculated tissue (Fig 1B). In susceptible pepper plants, PVX systematically spreads throughout the plant from the site of infection. Similarly, PVX::AvrPto was also expected to show systemic spread. However, the systemic movement of PVX::AvrPto is restricted in pepper and can only be detected in infected lower leaves. This is may be due to the localization of AvrPto by a Pto homolog in inoculated leaves, and thus resulting in the inhibition of systemic movement of the PVX::AvrPto. Therefore, signal for neither PVX antigen nor AvrPto was detected in the upper uninoculated leaves (Fig 1B, Lane 4).

Because all PVX-susceptible pepper genotypes appeared to recognize AvrPto, based on the appearance of characteristic necrotic local lesions, conventional genetic approaches that require differential host responses are not applicable. The availability of a mutant elicitor, *AvrPto*^I96T^ that specifically inhibits interaction with *Pto*, allowed an alternative approach. If a *Pto* homologous sequence from pepper encodes a functional gene product *in vivo* that is able to recognize AvrPto and if this recognition event is important in localizing PVX infection, then PVX vectors expressing *AvrPto*^I96T^ should spread systemically. When pepper seedlings were inoculated with pPVX::AvrPto^I96T^, chlorotic spots appeared on inoculated leaves that developed necrotic halos of approximately the same size as the necrotic local lesions observed in the incompatible interaction. These symptoms subsequently spread throughout the plant (Fig 1A panels V, VI). Systemic infection was confirmed by the presence of PVX antigen (data not shown) and hybridization of total RNA from upper uninoculated leaves with PVX- and *AvrPto*-homologous probes (Fig 1B).

Taken together, only PVX constructs expressing *AvrPto* were restricted to inoculated tissue in two pepper species. All other PVX constructs moved systemically, which is consistent with the hypothesis that the Pto homologs in pepper may also function to recognize AvrPto *in vivo*.

### Identification, classification and phylogenetics of Ptos and PLPKs

To identify the possible interactor of AvrPto, we performed a genome-wide analysis to search for tomato a Pto homolog in the CM334 pepper cultivar. To distinguish the Pto homologs from other classes of STK-domain containing proteins, a phylogenetic tree was constructed using Pto homologs from various plant species and various RLKs from *Arabidopsis* [42]. Only the region between subdomain I and XI of the STK domain was included in the phylogenetic tree. Pto homologs, including PLPKs, clearly formed a separate cluster from *Arabidopsis* RLKs and other protein kinase classes (S2 Fig). Based on sequence similarity and phylogenetic analysis, 25 Pto-like protein kinase genes were identified in the pepper genome (Table 1).

**Table 1:** Pto-like genes in pepper and other pepper genes related to Pto pathway.

Gene	Name	ID *	Chromosome	Position	Localization^†^	AA^#^
*Pto*	*PLPK1*	CA02g03150	2	39576898–39579564	PM	891
	*PLPK2*	CA02g04830	2	63491885–63494494	PM	872
	*PLPK3*	CA02g24540	2	160452307–160455046	PM	837
	*PLPK4*	CA02g24550	2	160455872–160458654	PM	780
	*PLPK5*	CA02g24560	2	160461053–160463982	PM	881
	*PLPK6*	CA02g24570	2	160468371–160471285	PM	880
	*PLPK7*	CA02g27190	2	164021808–164024365	PM	683
	*PLPK8*	CA03g12160	3	110449199–110451655	PM	821
	*PLPK9*	CA03g26270	3	235259494–235262043	PM	852
	*PLPK10*	CA03g29130	3	239669836–239672391	PM	854
	*PLPK11*	CA06g01780	6	8745719–8748214	PM	834
	*PLPK12*	CA06g08690	6	166977407–166980016	PM	873
	*PLPK13*	CA06g13920	6	203491113–203493602	PM	832
	*PLPK14*	CA07g16480	7	220808727–220811249	PM	840
	*PLPK15*	CA09g08930	9	101067855–101070542	PM	898
	*PLPK16*	CA09g11270	9	180794286–180796871	PM	864
	*PLPK17*	CA10g00440	10	523482–526010	PM	845
	*PLPK18*	CA10g08680	10	116218177–116220310	PM	865
	*PLPK19*	CA00g91560	PGAv.1.5.scaffold2195	5965–8481	PM	842
	*PLPK20*	CA00g74940	PGAv.1.5.scaffold1602	89324–91768	PM	817
	*PLPK21*	CA00g66280	PGAv.1.5.scaffold1447	110520–113048	PM	845
	*PLPK22*	CA00g71970	PGAv.1.5.scaffold1544	153054–155699	PM	884
	*PLPK23*	CA00g68580	PGAv.1.5.scaffold1481	174625–177213	EPM	865
	*PLPK24*	CA00g56150	PGAv.1.5.scaffold1280	474406–477018	PM	873
	*PLPK25*	CA00g56160	PGAv.1.5.scaffold1280	478736–481369	PM	881
*Pti*	*Pti1*.*1*	CA03g02330	3	5111614–5122173	PM	399
	*Pti1*.*2*	CA05g15640	5	219544242–219547310	PM	396
	*Pti1*.*3*	CA12g20440	12	229696215–229700013	PM	354
	*Pti1*.*4*	CA00g35470	PGAv.1.5.scaffold960	156795–159526	PM	369
	*Pti4*	CA05g13540	5	207246767–207247186	NL	139
	*Pti5*	CA02g04360	2	60559197–60559739	NL	180
	*Pti6*.*1*	CA06g11050	6	190499179–190500481	NL	168
	*Pti6*.*2*	CA00g30930	PGAv.1.5.scaffold874	960106–960858	NL	250
*Prf*	*Prf1*.*1*	CA04g03860	4	14555496–14559641	CP	1219
	*Prf1*.*2*	CA11g01790	11	4570265–4573977	CP	1128
	*Prf1*.*3*	CA11g06970	11	53883501–53886421	CP	749
	*Prf1*.*4*	CA00g31190	PGAv.1.5.scaffold875	354725–359092	CP	1206

PM; Plasma-membrane, EPM; Extracellular plasma-membrane, CP; Cytoplasm, NL; Nuclear

* Pepper genome database v1.5

† Predicted subcellular localization

# predicted amino acid length

**Fig 2:**
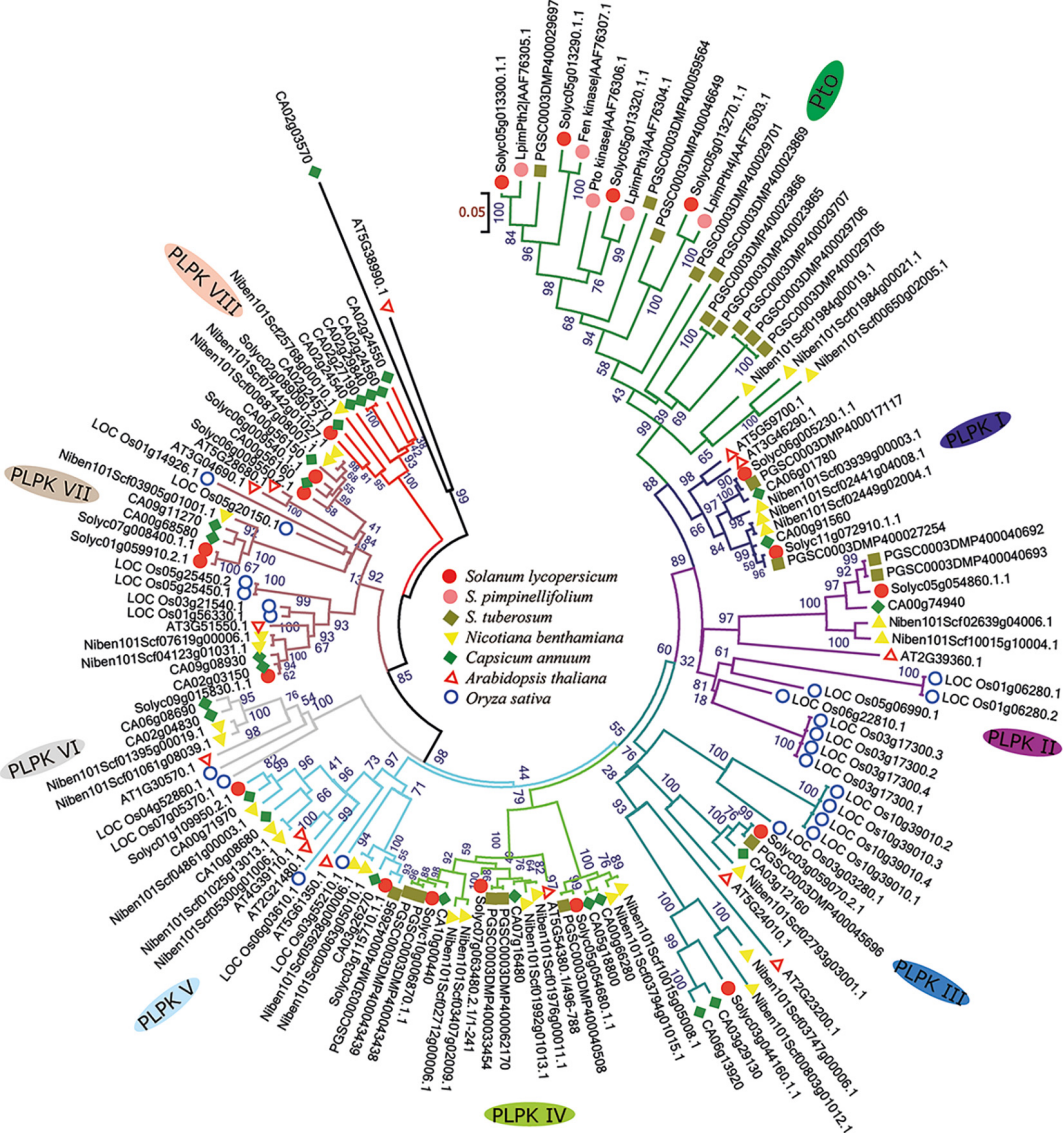
Phylogenetic analysis of tomato Pto paralogs and PLPKs. A rooted phylogenetic tree was constructed using the predicted pepper PLPK sequences and full-length Pto proteins from tomato, *N*. *benthamiana*, potato, *Arabidopsis* and rice. The phylogenetic tree was constructed using the NJ method (1000 bootstrap replicates) as implemented in the MEGA 6.0 software. The name is indicated next to each subclass. Pto-like proteins from various plant species are classified into two main classes, Pto and PLPKs. PLPKs are further divided into eight subclasses (PLPK I-PLKPK VIII).

To examine the evolutionary relationships between Pto and PLPK homologs, and to classify the pepper PLPKs, a rooted phylogenetic tree was constructed using the predicted pepper PLPK sequences and full-length Pto and PLPK homologs from various plant species, such as tomato, *N*. *benthamiana*, potato, *Arabidopsis* and rice (Fig 2). Phylogenetic analysis showed the existence of two distinct clusters of Pto homologs, Pto and PLPK (Fig 2). The Pto clade is specific to Solanaceae species, and within this clade homologs of tomato Ptos are clustered together. However, no Pto clade members were found in pepper, suggesting that the origin of Ptos occurred during the early evolution of Solanaceae sublineages. The pepper PLPKs further separated into eight subclasses (PLPK I to PLPK VIII). In subclass PLPK I and PLPK IV, PLPKs from various dicot species were clustered together, whereas in subclasses PLPK II, PLPK III, PLPK V, PLPK VI, and PLPK VII, both dicot and monocot PLPK members were present. However, in subclass PLPK II and PLPK III monocot and dicots formed distinct clades. At least one *Arabidopsis* PLPK member was observed in all PLPK subclasses, with the exception of subclass PLPK VIII, which is specific to the Solanaceae family. Overall, the phylogenetic analysis showed that pepper PLPKs cluster in a similar pattern to other dicots. Moreover, PLPK members from rice can be found among dicot members throughout the phylogenetic tree, implying that PLPKs have evolved in the common ancestors of monocots and dicots.

### Phylogenetic analysis of pepper PLPKs

**Fig 3:**
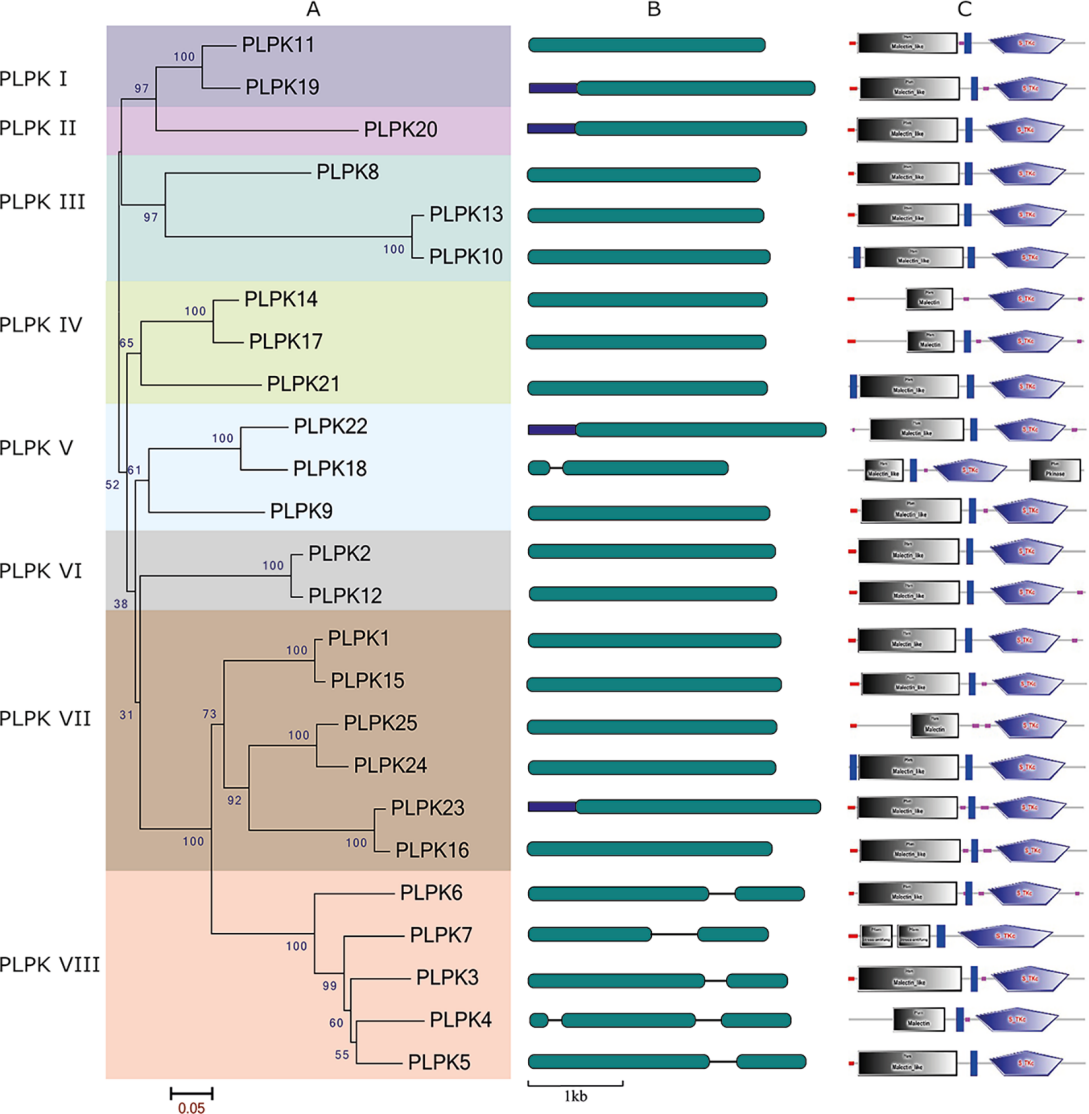
Phylogenetic analysis of pepper PLPKs. A: A rooted phylogenetic tree was constructed using the predicted pepper PLPK sequences. The phylogenetic tree was constructed using the NJ method (1000 bootstrap replicates) as implemented in the MEGA 6.0 software. The name of each subclass is indicated. Phylogenetic analysis showed distinct clustering of PLPKs into eight subclasses (PLPK I–PLKPK VIII). B: Exon-intron structures of pepper *PLPK* genes. UTRs and exons are shown as blue and dark cyan boxes, respectively, and introns are shown as black lines. C: Structural and functional domains identified by the SMART program. Most pepper PLPKs contained a signal peptide region (red box), a malectin-like region, a transmembrane (TM) region (blue box), and a STK domain. PLPK18, which belongs to the PLPK V class, has an additional kinase domain at the C-terminus. PLPK7, which belongs to the PLPK VIII subclass, includes two stress-antifung domains at the N-terminus. In addition, low complexity regions (violet box) were present in several PLPKs.

To determine the phylogenetic relationships between pepper PLPKs, a separate phylogenetic tree was constructed with pepper PLPKs alone. From this phylogenetic analysis, it appears that pepper PLPKs can also be classified into the eight subclasses PLPK I to PLPK VIII (Fig 3). Subclasses PLPK I and PLPK VI include two members, subclasses PLPK III and PLPK V include three members, and subclasses PLPK II, PLPK IV, PLPK VII, and PLPK VIII include one, three, six, and five members, respectively.

### Comparative phylogenetic analysis of pepper PLPKs and tomato Ptos and PLPKs

To determine the phylogenetic relationships between pepper and tomato Pto homologs, a phylogenetic tree was generated (S3 Fig), which showed that Pto class members are not present in pepper. All PLPK homologs showed similar clustering patterns in both pepper and tomato with the exception of subclass PLPK VI, which was not represented in tomato. The number of PLPKs observed in the subclasses PLPK I, PLPK II, and PLPK IV was the same in pepper and tomato i.e. 1, 2, and 3, respectively. In each of the PLPK subclasses PLPK III and PLPK V, 2 and 3 members were observed in tomato and pepper, respectively. In subclass PLPK VII, 5 and 6 members were observed in tomato and pepper, respectively. In subclass PLPK VIII, five members were found in pepper, whereas only one PLPK member was found in tomato. These observations suggest that there has been sublineage expansion and diversification of Ptos and PLPKs in plants of the Solanaceae.

### Chromosomal distribution and structure of pepper PLPK and Pto pathway genes

We named the pepper *PLPK* genes as *PLPK1* to *PLPK25*, based on their order on chromosomes (S4A Fig). The details of the proposed *PLPK* gene names in pepper and their IDs are presented in Table 1. Seven out of 25 *PLPK* genes were not assigned to any chromosome. The remaining 18 *PLPK* genes were localized to six chromosomes. The number of the genes on the chromosomes varied greatly from none on Chr1, Chr4, Chr5, Chr8, and Chr12 to a maximum of seven on Chr2. Chr3 and Chr6 each had three *PLPK* genes. Chr9 and Chr10 each showed two *PLPK* genes, whereas a single *PLPK* was localized to Chr7 (S4A Fig). Based on linkage mapping analysis, out of seven *PLPK* genes, which were not assigned to any chromosome, at least one *PLPK* gene could be assigned to Chr12, and two PLPKs (*HPto-a* and *HPto-d*) which are collinear with the *Prf* homologs (*Prf1*.*2* (*HPrf-c*) and *Prf1*.*3* (*HPrf-b*)), could be assigned to Chr11 (S4B Fig). *HPto-a* and *HPrf-b* (*Prf1*.*3*) were mapped to the corresponding position of the tomato *Pto* clade, suggesting that *HPto-a* is an ortholog of tomato Pto. However, in contrast to tomato *Prf*, which is embedded in the *Pto* cluster, *HPrf-b* was located approximately 2 cM from *HPto-a*.

Among the four *Prf* homologs, *Prf1*.*1* was located on Chr4, *Prf1*.*2* and *Prf1*.*3* were located on Chr11, and *Prf1*.*4* could not be associated with any chromosome. Among the four *Pti1* homologs, *Pti1*.*1*, *Pti1*.*2* (*HPti1-a*), and *Pti1*.*3* were located on Chr3, Chr5, and Chr12, respectively, and *Pti1*.*4* (*Hpti1-b*) could be located to Chr5 based on the linkage map (S4B Fig). The two *Pti1* homologs, *HPti1-a* and *Hpti1-b*, that were detected in pepper, showed synteny with tomato Chr5. *Pti4*, *Pti5*, and *Pti6*.*1* were located on Chr5, Chr2, and Chr6, respectively. *Pti6*.*2* was not located to any chromosome.

Exon-intron structure analysis showed that most of the pepper *PLPK* genes are intron less with some exceptions; *PLPK3*, *PLPK5*, *PLPK6*, *PLPK7*, and *PLPK18* genes showed one intron, and *PLPK4* gene showed two introns (Fig 3B). In contrast, pepper *Prf* genes showed three to six introns with different lengths and positions (S5 Fig). Similarly, *Pti* genes were also found to be highly divergent and showed a complex structure with zero to seven introns having variable distributions (S5 Fig).

### Conserved motif analysis of pepper PLPKs

**Fig 4:**
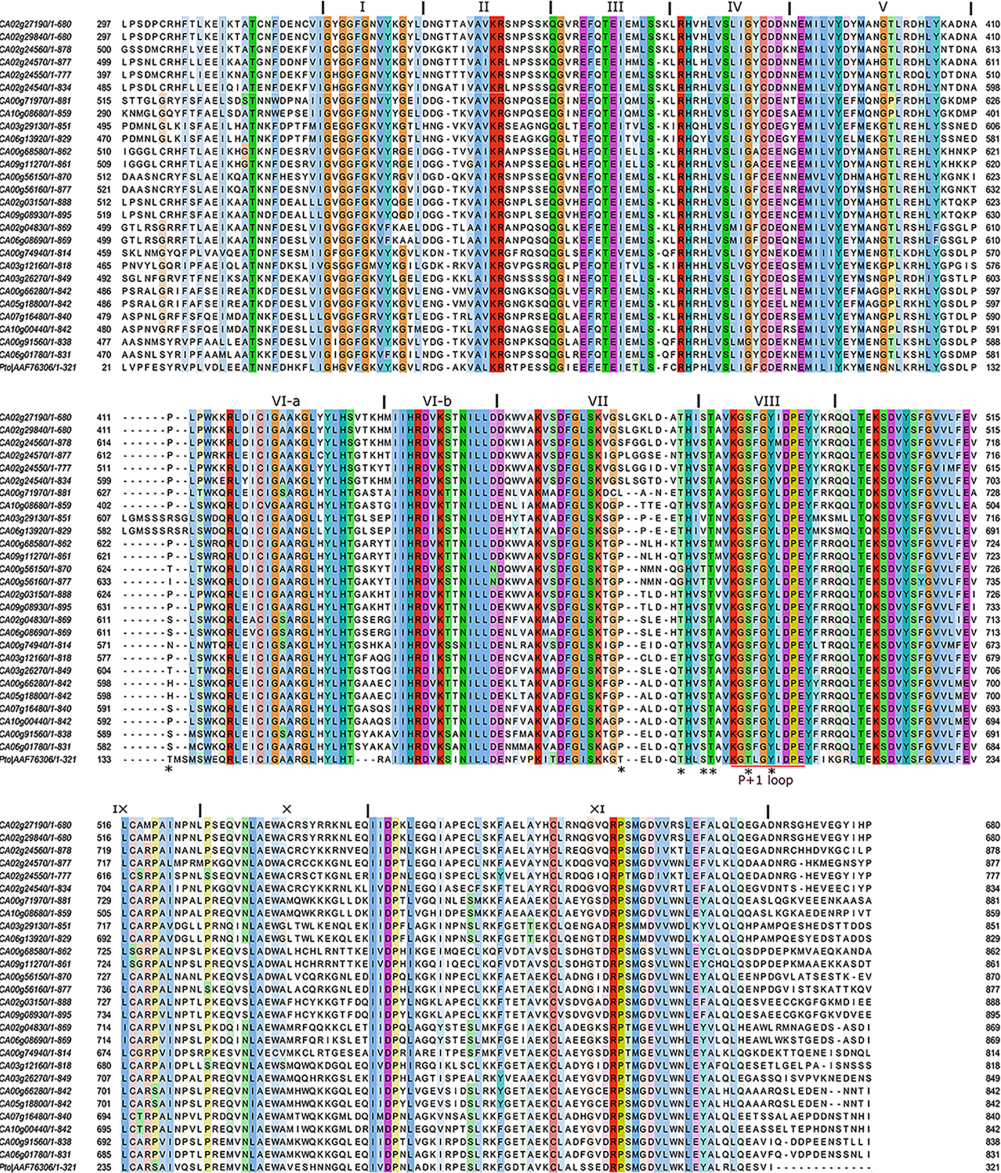
Multiple sequence alignments of the STK region. The STK domains were aligned using the Clustal Omega program, and the alignments were displayed in the "ClustalX" color mode available in JalView 2.8. The conserved domains I through XI are indicated in the figure. Autophophorylation sites are indicated with asterisks.

A multiple sequence alignment of the predicted amino acid sequences of the 25 pepper PLPKs and the corresponding region of tomato Pto was performed using the Clustal Omega program. The predicted length of the pepper PLPK sequences varied from 683 to 898 amino acids. Analysis of the predicted amino acid sequences of the PLPKs from pepper showed 54 to 71% sequence identity with tomato Pto. Within the pepper PLPK phylogenic groups, the amino acid sequence identity varied from 35 to 96%. All pepper PLPKs showed a highly conserved STK domain of approximately 275 amino acids (Fig 4). Each of the eight subclasses contains the conserved amino acid residues found in subdomain I through XI of the STK domains that are found in most of the plants. Alignment of amino acid sequences revealed several characteristic features of PLPKs that are highly conserved in the Pto homologs, such as the STK domain, the presence of the activation domain located between subdomains VII and VIII, and the internal P+1 loop site, which is responsible for the specific binding of AvrPto effectors [50]. The STK domain also contained several dispersed variable amino acid sites. In addition, three of the four autophosphorylation sites (Ser or Thr) in the activation domain of Pto [51] are conserved in the corresponding region of all pepper PLPKs (Fig 4). Mutations at the highly conserved Val55 and His94 positions were reported to disrupt the Pto-AvrPto interaction in yeast and inhibit the Pto-mediated resistance [52].

We used the SMART program to confirm the structural features of the PLPKs and found that most of the PLPKs contained four domains; a signal peptide region, a malectin-like region, a transmembrane region, and the STK domain (Fig 3C). By contrast, tomato Pto contained only the STK domain. PLPK18, a member of class PLPK V, harbored an additional kinase domain at its C-terminus. PLPL7, a PLPK VIII class member, included two stress-antifung domains at its N-terminus. In addition, several of the pepper PLPKs showed low complexity regions. The protein subcellular localization prediction tool, EuLoc [45], showed that all pepper PLPKs proteins are predicted to localize to the plasma membrane. All Pti1 proteins were predicted to localize to the plasma membrane as well, whereas Pti4, Pti5, and Pti6 were predicted to localize to the nucleus, and the Prfs were predicted to localize to the cytoplasm.

To identify conserved Pto autophosphorylation sites and other critical residues in the activation domain of the PLPKs from pepper, multiple sequence alignment analysis was performed including the tomato Pto sequence (Fig 4). Three of the five Pto autophosphorylation sites [51], Thr195, Ser198, and Thr199 were highly conserved in pepper PLPKs with a few exceptions; Thr195 was replaced by Gly in PLPK24 and PLPK25, and Ser198 was replaced by Val in PLPK10 and PLPK13, and Thr in PLPK24 and PLPK25 sequences. Ser198 is required for the AvrPto-Pto-mediated hypersensitive response, and was highly conserved in pepper PLPKs. The Pto autophosphorylation site, Thr190, was replaced by a Pro in the majority of the pepper PLPKs. In some of the PLPKs, such as PLPK3, PLPK4, PLPK5, and PLPK7, a Ser was replaced by Thr, creating an alternative phosphorylation site. In PLPK22, Thr190 was replaced by Leu. Two other Pto phosphorylation residues, Thr204 and Tyr207, are crucial for Pto function but are not autophosphorylated *in vitro* [14, 53]. Both these Pto phosphorylation residues are highly conserved; however, the Thr204 was replaced by a Ser in all pepper PLPKs.

### Analysis of selection pressure

To examine whether the *Pto* and *PLPK* gene classes have been subjected to adaptive evolution following gene duplication, an analysis of variation in selective pressure was carried out. A codon-based multiple sequence alignment was analyzed through the use of various models that were accessed through the Datamonkey web server for the detection of selective pressure [47]. The alignment was also analyzed with GARD to rule out recombination break points, and this analysis showed no evidence of any recombination events. To identify past selection on individual codons, the rate of nonsynonymous (dN) and synonymous (dS) substitutions and the dN/dS (ω) ratio at each codon site were calculated with various Codon-based maximum-likelihood methods (FEL, IFEL, and SLAC). Interestingly, no evidence of positive selection on any site was shown by these methods (Table 2 and S6 Fig). The majority of sites showed evidence of negative/purifying selection, while between 13 and 22 sites (depending on which model was used) were found to be evolving at a neutral rate. Interestingly, the mixed effects model of evolution (MEME) identified 16 amino acid sites (S6 Fig) as being under episodic positive selection (0.1 significance level). Among these, two amino acid sites were located at the N-terminus of the STK domain, one amino acid site each in the II, III, VI-a, VI-b, and VII subdomains, two sites each in the VIII, IX, and X subdomains, and three sites in the XI subdomain. These results suggest that, in addition to purifying selection, positive selection has played an important role in evolution of Pto homologs in plants, and thus in the evolution of adaptive disease resistance.

**Table 2:** Positive selection analysis of the STK domain of *Pto-like* genes from various plants.

Model	Neutrally evolving sites	Positively evolving sites	Negatively evolving sites
FEL	13	0	262
IFEL	22	0	253
SLAC	19	0	256

Positive selection analysis was performed by various models (at p value 0.1) as implemented in the Datamonkey web server

### Expression profile of Pto pathway genes

**Fig 5:**
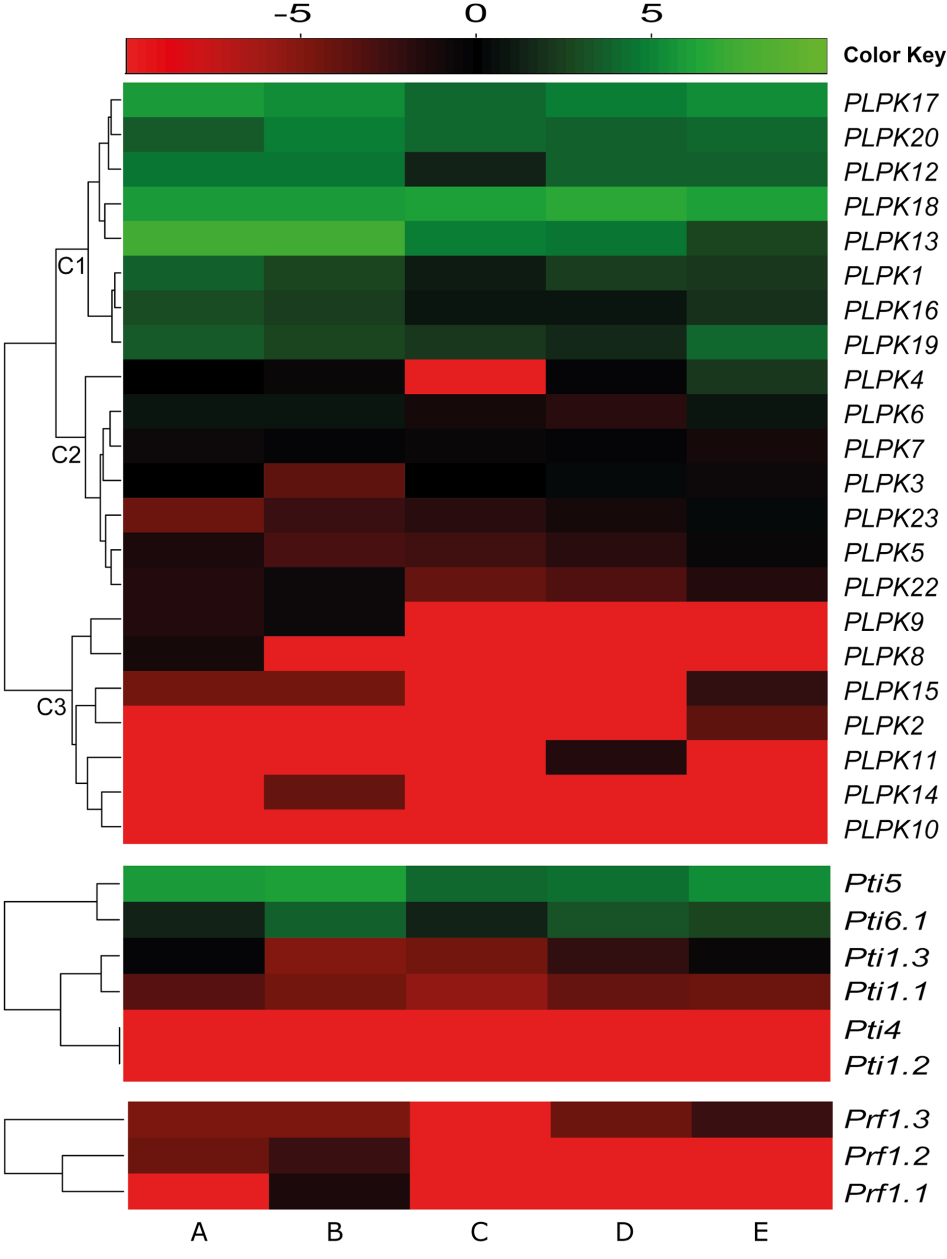
Expression profiles of *PLPK* genes from five different pepper accessions. A heatmap was generated using log2 transformed RPKM expression values. The color bar at the top represents log2 expression values. The green, black and red colors represent high, medium, and low expression levels. A; PI260429, B; PI152225, C; PI201234, D; YCM334, E; Aji Dulce.

Expression profiles of Pto pathway genes were generated using previously published RNA-seq data from uniformly grown five different pepper accessions [48]. To analyze the expression profiles of pepper *PLPK* genes among different accessions, heatmap and hierarchical clustering based on the mean-centered log (base 2) transformed RPKM values of the *PLPK* genes were generated (Fig 5). Based on expression pattern, pepper *PLPK* genes clustered into three groups; C1, C2, and C3 (Fig 5). In group C1, *PLPK1*, *PLPK12*-*PLPK13*, and *PLPK16*-*PLPK20*, which were relatively highly expressed, clustered together. Interestingly, at least one gene from each pepper PLPK subclass was observed in C1, with the exception of subclass VIII. All of the subclass VIII members (*PLPK3-PLPK7*), and *PLPK22* and *PLPK23*, which belong to subclass V and VII respectively, were clustered in group C2 and were expressed at moderate levels. The pepper *PLPK* genes *PLPK2*, *PLPK8*-*PLPK11*, and *PLPK14*-*PLPK15* were clustered in group C3, and were expressed at low levels. Interestingly, at least one gene from each subclass, with the exception of subclasses PLPK II and PLPK VIII, was observed in group C3. Many of the pepper Pto pathway genes, including *Pti* and *Prf*, also showed moderate to low expression levels. In general, plant *R* genes exhibit a low level of constitutive expression in either infected or uninfected plants, which is in accordance with their general role in pathogen recognition [54, 55]. Taken together, the expression profiles of Pto pathway genes in the five pepper genotypes (*C*. *annuum* ‘PI201234’ and ‘YCM334’, *C*. *chinense* ‘Aji Dulce’ and ‘PI152225’, and *C*. *chacoense* ‘PI260429’) were very similar (Fig 5), indicating a highly conserved gene regulatory mechanism for Pto signaling in pepper species.

## Discussion

In tomato, the *Pto* locus consists of a small cluster of five *Pto* homologs present on chromosome 5, that possibly evolved through sequential gene duplications and deletions [10, 34]. The *Pto* locus and its gene-for-gene role in AvrPto recognition specificity are believed to be conserved in Solanaceae species [19, 54]. However, the recognition specificity of Pto is unknown in a number of Solanaceae species, including pepper and potato. Despite the recognition of AvrPto by many plant species, it is unknown whether the recognition is mediated by Pto-like kinases. In addition, no functional Pto-like proteins that recognize AvrPto have been identified in other plant species, with the exception of tomato and tobacco. Our results using a PVX-based system with pepper provide evidence for *in vivo* recognition of AvrPto. However, we cannot rule out the possibility that the recognition of AvrPto in pepper plants may be due to Pto-like genes and/or other RLKs, until our results are verified using reverse genetic approaches. It is well established that Pto, through interaction with the NB-LRR Prf, mediates recognition of AvrPto/AvrPtoB in tomato [56]. However, the targets of AvrPto/AvrPtoB appear to be the kinase domains of various RLKs [57–60] rather than the Pto STK domain; Pto is being used as a molecular bait by Prf to interact with pathogen effector proteins [61, 62]. AvrPto/AvrPtoB effectors interact with several plant molecules, such as *Arabidopsis* GTPase RabE [63, 64] and the kinase domains of several RLKs, such as CERK1, BAK1, EFR1, and FLS2 [57–60, 65].

To identify possible *AvrPto*-interacting tomato *Pto* homologs in pepper, we carried out a genome-wide analysis, which resulted in the identification of 25 full-length *Pto-like* (*PLPK*) genes. Most *R* genes are members of multigene families, and occur as clusters in plant genomes. Gene duplication and diversification are thought to be responsible for the evolutionary expansion of the *R* gene clusters [5]. In agreement with this, the present and previous phylogenetic analyses of *Pto* homologs from a number of plant species revealed several subclasses of *Pto* homologs [27, 30, 32]. Interestingly, no Pto clade members were found in pepper. Homology searches against the three different pepper genome databases “CM334”, “Chiltepin”, and “Zunla-1” (http://cab.pepper.snu.ac.kr/), and pepper expressed sequence tags (ESTs) at NCBI, confirmed the absence of tomato Pto homologs (Pto clade) in the pepper genome. Wan *et al*. [66] failed to amplify true tomato *Pto* homologs in pepper when using primers designed against the conserved regions of *Pto* genes. Furthermore, no homologs of the tomato Pto class were detected *R* gene mapping studies in pepper [67]. With the exception of the Pto clade, Solanaceae PLPKs from pepper and other plant species are well-distributed among the branches of the phylogenetic tree, suggesting that PLPKs arose from common ancestral genes prior to the divergence of monocots and dicots. The absence of tomato *Pto* homologs in pepper further suggests Solanaceae sublineage-specific expansion of the Pto clade. This observation also suggests that the role of *Pto* genes in pathogen recognition and disease resistance might have evolved during early evolution of Solanaceae lineages [27, 35] or, alternatively, has been lost from pepper.

The underlying mechanism that could result in the loss of the Pto clade in pepper and some tomato genotypes [34, 35] is not clearly understood. Although some cultivated tomato, *S*. *lycopersicum*, varieties show *avrPto-*specific *Pto*-mediated *Pst* resistance, the resistance was introgressed from *S*. *pimpinellifolium* [10, 36]. Two possible reasons have been suggested to explain the fact that Pto recognition specificity has been retained in some tomato species and entirely lost in closely related species. First, during the domestication of wild *S*. *lycopersicum*, an intense genetic bottleneck might have occurred and resulted in the selection of those *S*. *lycopersicum* lines that lacked the *Pto* gene [35]. Second, fitness costs associated with the *Pto* locus in the absence of *Pst* with *avrPto* gene expression might have resulted in selection against *S*. *lycopersicum* lines containing the *Pto* gene [35]. However, near-isogenic lines of *S*. *lycopersicum* introgressed lines with and without the *Pto* locus showed no evidence of a fitness cost associated with *Pst* resistance [35, 68], although a deleterious effect of the *Pto* locus may be difficult to determine experimentally [35].

Despite extensive studies in tomato, the functional specificity of *Pto* homologs in other Solanaceae species is not clearly understood. *Pto* paralogs share 78 to 91% nucleotide identity with tomato *Pto*, and despite having functional protein kinase activity, none can interact with AvrPto and AvrPtoB effectors [12, 69]. Gene duplication and subsequent diversification of the *Pto* gene family presumably have led to alternative recognition specificities [70]. Pto and Pto homologs vary only in a few amino acid residues, which could cause protein conformational changes that result in altered ability to physically interact with other proteins [36]. The *Fen* gene, which is one of the five *Pto* paralogs, is 87% identical to *Pto* and participates in the same signaling events leading to HR. However, it is activated by a different signal [70, 71]. By contrast, LhirPto shows 97% sequence identity with only 17 amino acid variations that distinguish it from Pto, and yet LhirPto does not abolish Pto disease resistance [35]. These observations further indicate that there has been a selective pressure to retain Pto-AvrPto recognition specificity [72].

Diversifying selection or adaptive selection are presumed to play a key role in the evolution of *R* genes. Adaptive selection is considered the main force that drives evolution of gene regions that function in host-pathogen recognition [5, 73]. Interestingly, purifying selection appeared to be the predominant selective pressure on genes that contain STK domains to maintain their ancestral state [32]. However, in the present study, episodic positive selection was identified in the background, suggesting a possible change in selection pressure during evolution. It has been suggested that adaptive evolution often involves episodic bursts of selection localized to a few sites in a gene, and may affect only a subset of lineages in phylogeny [74]. In agreement with this, MEME analysis showed that some codon sites are under episodic positive selection. The activation domain of Pto, the region between amino acids 182 and 211, plays an important role in AvrPto recognition [27]. Interestingly, our results showed that two positions, Ser198 and Ile208, were subjected to episodic positive selection, implying that they play an important role in adoptive evolution. Recent data show that *N*. *benthamiana* plants, which have been used as negative controls for the Pto/AvrPto response, can also recognize AvrPto in a gene-for-gene manner, and that this recognition is dependent on Prf [19]. Recognition of AvrPto resulting in plant resistance has also been observed in distant taxa, for example in soybean [28]. These observations further suggest that, in addition to the purifying and balancing selection [72], adaptive selection could also have influenced the evolution of the *Pto* locus.

In summary, nonhost disease response associated with recognition of the AvrPto effector from *Pst* was observed in pepper genotypes. Recognition of AvrPto was possibly mediated by Pto homologs or other RLKs. We identified 25 pepper *PLPK* genes through genome-wide analysis and found that they were divided into eight phylogenetic subclasses. The Pto clade represents a gene family of recent origin in Solanaceae species, but the PLPKs represent diverged genes with an ancient common origin. Tomato *Pto* paralogs are not found in the pepper genome, suggesting that Pto family genes have undergone different evolutionary processes towards adaptive evolution to *Pst* resistance. Pepper PLPKs were characterized by the presence of highly conserved STK domains. The presence of carbohydrate binding malectin-like domains in the PLPKs suggests a possible role in sugar sensing and disease resistance signaling pathways [75]. Similar expression profiles of Pto pathway genes was observed in all five pepper genotypes. These observations suggest *PLPK* genes with highly conserved functional and structural domains share a highly conserved gene regulatory mechanism associated with the Pto signaling pathway among the pepper genotypes. Further studies on Pto and PLPKs, and their interaction with AvrPto or other effectors will provide more insight in the process of plant-pathogen coevolution. The present genome-wide analyses of pepper *PLPK* genes provide an important genetic resource for further functional studies to unravel the molecular basis of nonhost disease resistance in pepper.

## Supporting Information

S1 Fig Pepper genotypes challenged with PVX virus. 234, Habanero, RNaky, and ECW plants showed systemic symptoms of PVX infection, whereas CM334 and Perennial plants showed no symptoms of PVX infection. Images were photographed 14 dpi.
(TIF)

S2 Fig Molecular phylogenetic analysis of tomato Pto proteins, Pto-like proteins and other classes of plant protein kinases. Multiple sequence alignment was generated with Clustal Omega. The phylogenetic relationships were inferred using the NJ method (1000 bootstrap replications) as implemented in the MEGA 6.0 software. Pto proteins/Pto-like proteins, RLKs and other classes of plant protein kinases clearly formed three different clades. The numbers above the branches indicate bootstrap values.
(TIF)

S3 Fig Comparative phylogenetic analysis of tomato and pepper Ptos and PLPKs. The phylogenetic tree was constructed using the NJ method (1000 bootstrap replicates) as implemented in the MEGA 6.0 software. The name of each subclass is indicated.
(TIF)

S4 Fig **Pepper *PLPK* gene distribution and linkage map** A: The chromosome number is indicated at the top of each chromosome. B: Genetic linkage map containing the *Pto* pathway genes. Chromosomes 2, 5, 6, 9, 11, and 12 are from the map based on an F2 mapping population from *C. annuum* and *C. chinense* [40]. Framework markers are shown on each linkage group in black and the *Pto* pathway genes are shown in red.
(TIF)

S5 Fig Exon-intron structures of pepper *Prf* and *Pti* genes. (TIF)

S6 Fig Positive selection analysis of STK domain of *Pto* and *PLPK* genes. Positive selection analysis was performed by various models as implemented in the Datamonkey webserver (at *p* value 0.1) and mapped on the alignment.–and n indicates sites under negative and neutral selection pressure, respectively. Sites undergoing episodic selection pressure are indicated with “Ep”.
(PDF)
