# Supplementary figures and images for: Genome-Wide Analysis and Evolution of the Pto-Like Protein Kinase (PLPK) Gene Family in Pepper
Source: PLoS One. 2016 Aug 18;11(8):e0161545. doi: 10.1371/journal.pone.0161545 (PMC4990186; doi:10.1371/journal.pone.0161545)

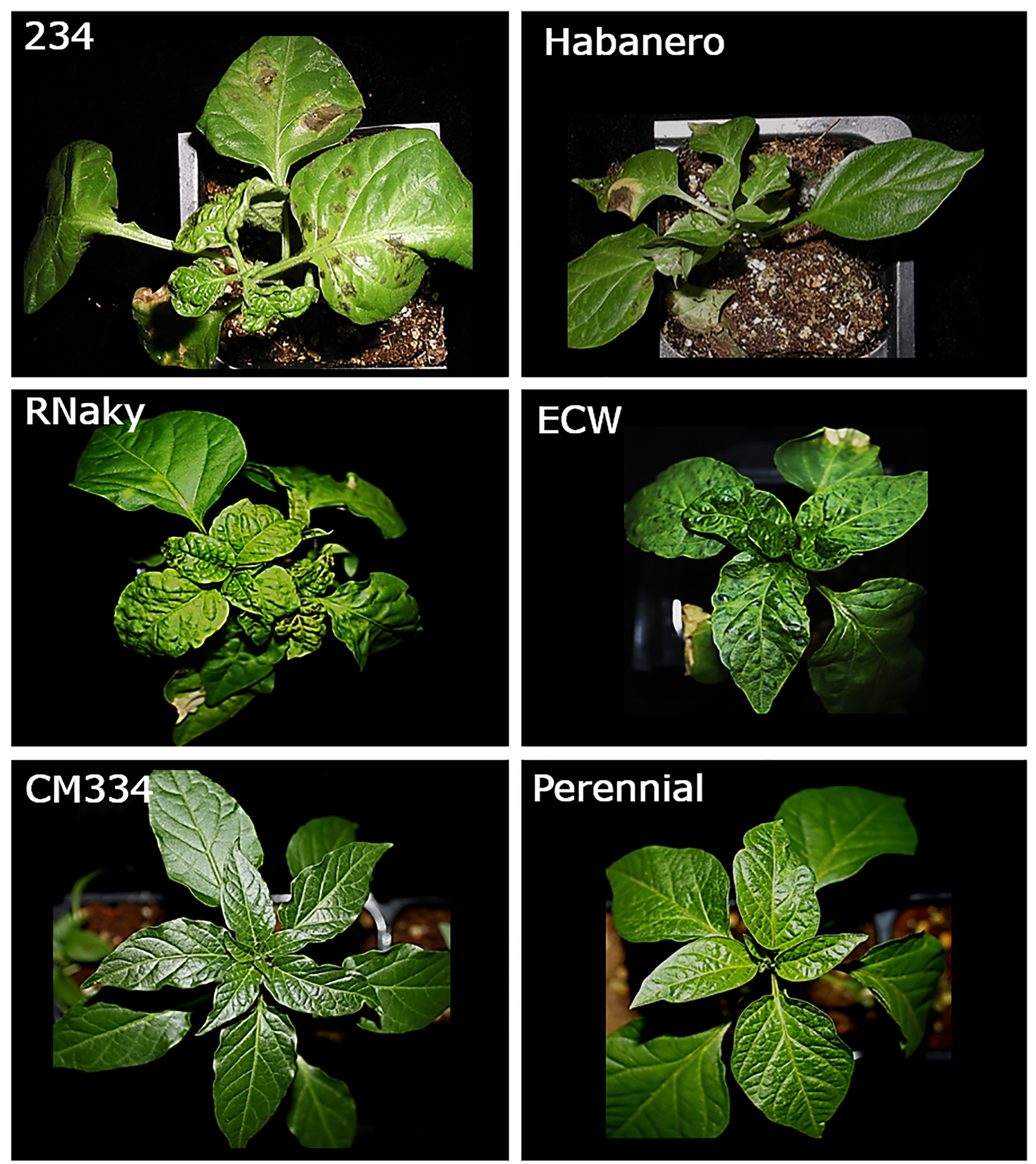

Supplement: S1 Fig — 234, Habanero, RNaky, and ECW plants showed systemic symptoms of PVX infection, whereas CM334 and Perennial plants showed no symptoms of PVX infection. Images were photographed 14 dpi. (TIF) [file pone.0161545.s001.tif]

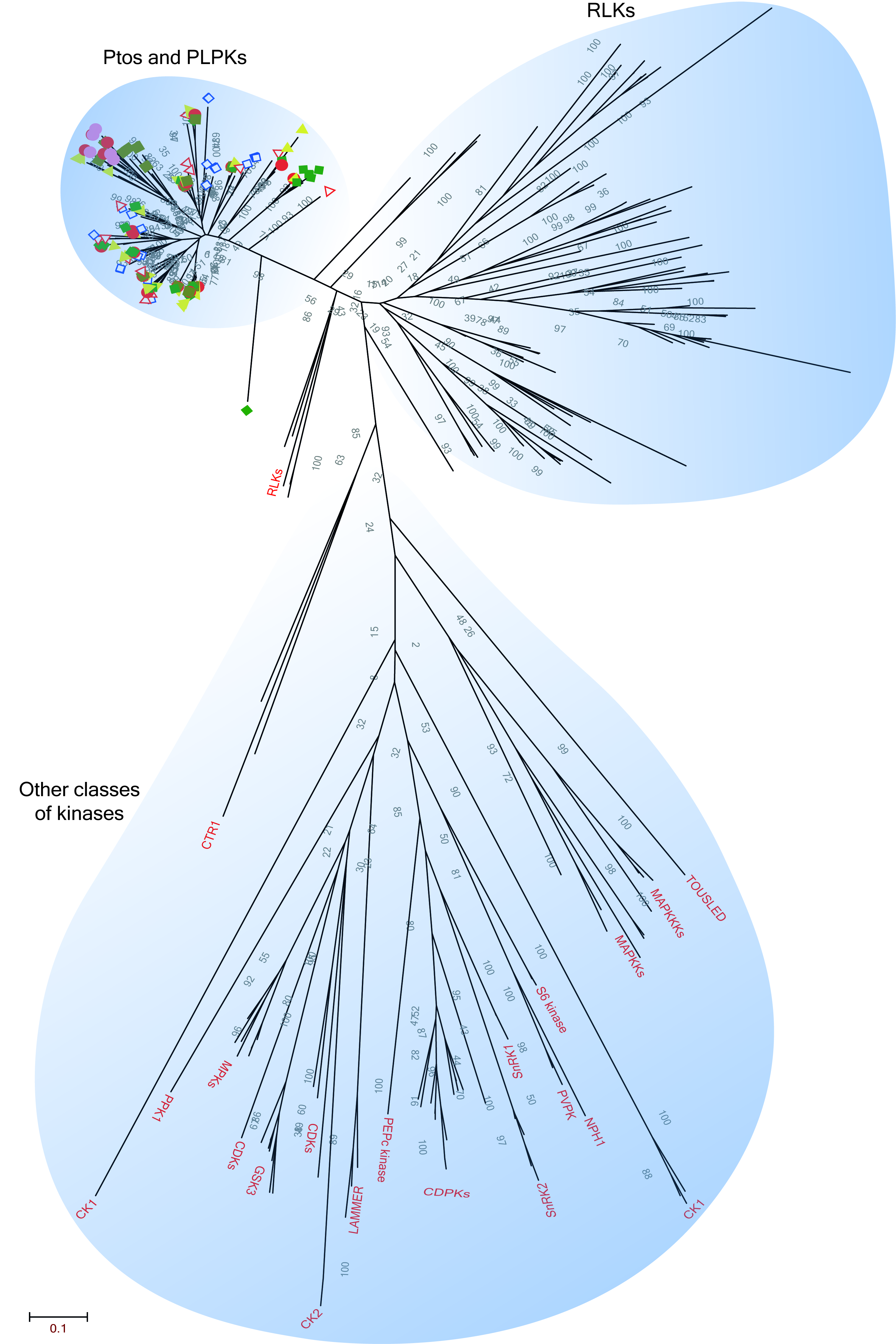

Supplement: S2 Fig — Multiple sequence alignment was generated with Clustal Omega. The phylogenetic relationships were inferred using the NJ method (1000 bootstrap replications) as implemented in the MEGA 6.0 software. Pto proteins/Pto-like proteins, RLKs and other classes of plant protein kinases clearly formed three different clades. The numbers above the branches indicate bootstrap values. (TIF) [file pone.0161545.s002.tif]

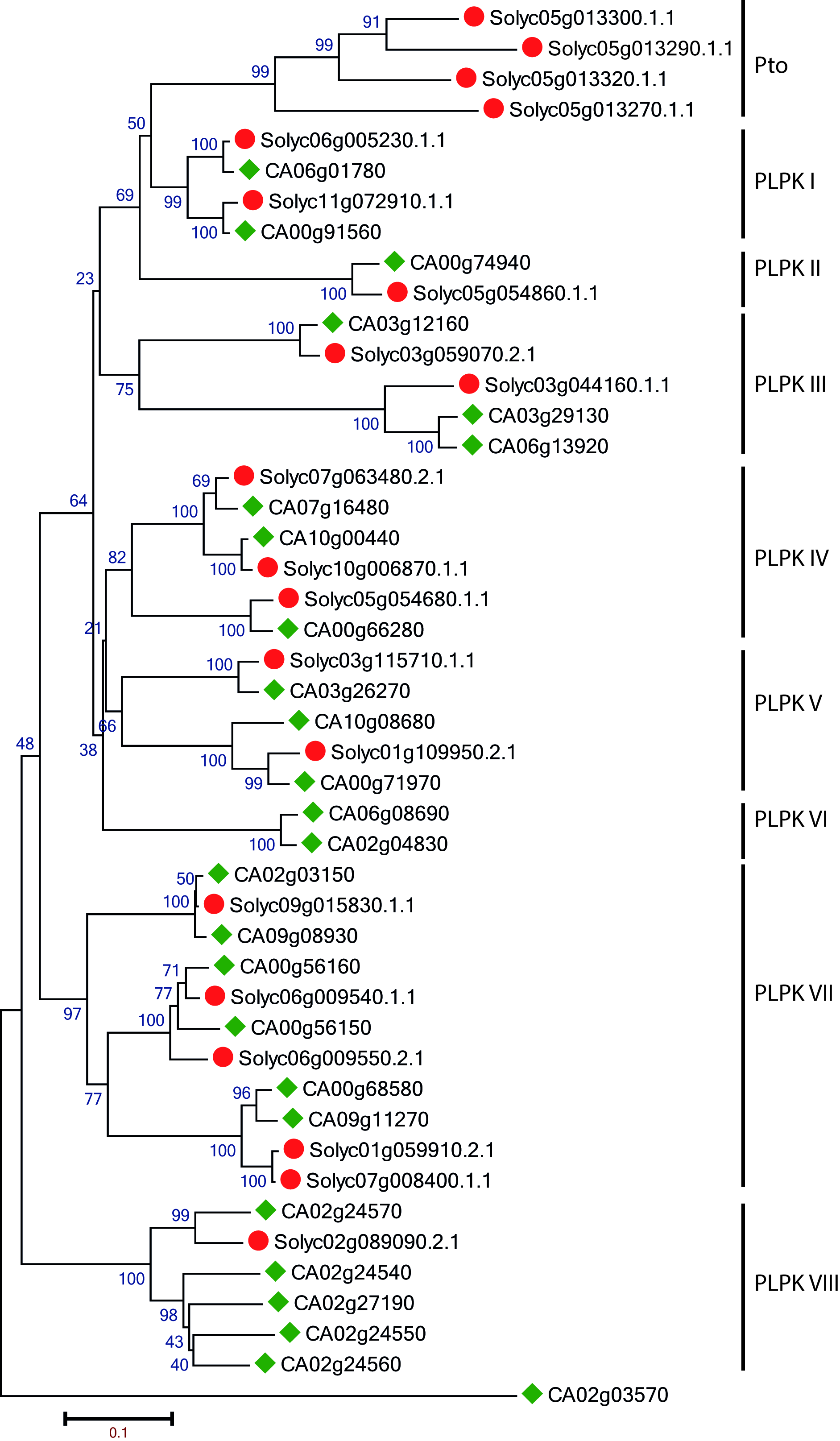

Supplement: S3 Fig — The phylogenetic tree was constructed using the NJ method (1000 bootstrap replicates) as implemented in the MEGA 6.0 software. The name of each subclass is indicated. (TIF) [file pone.0161545.s003.tif]

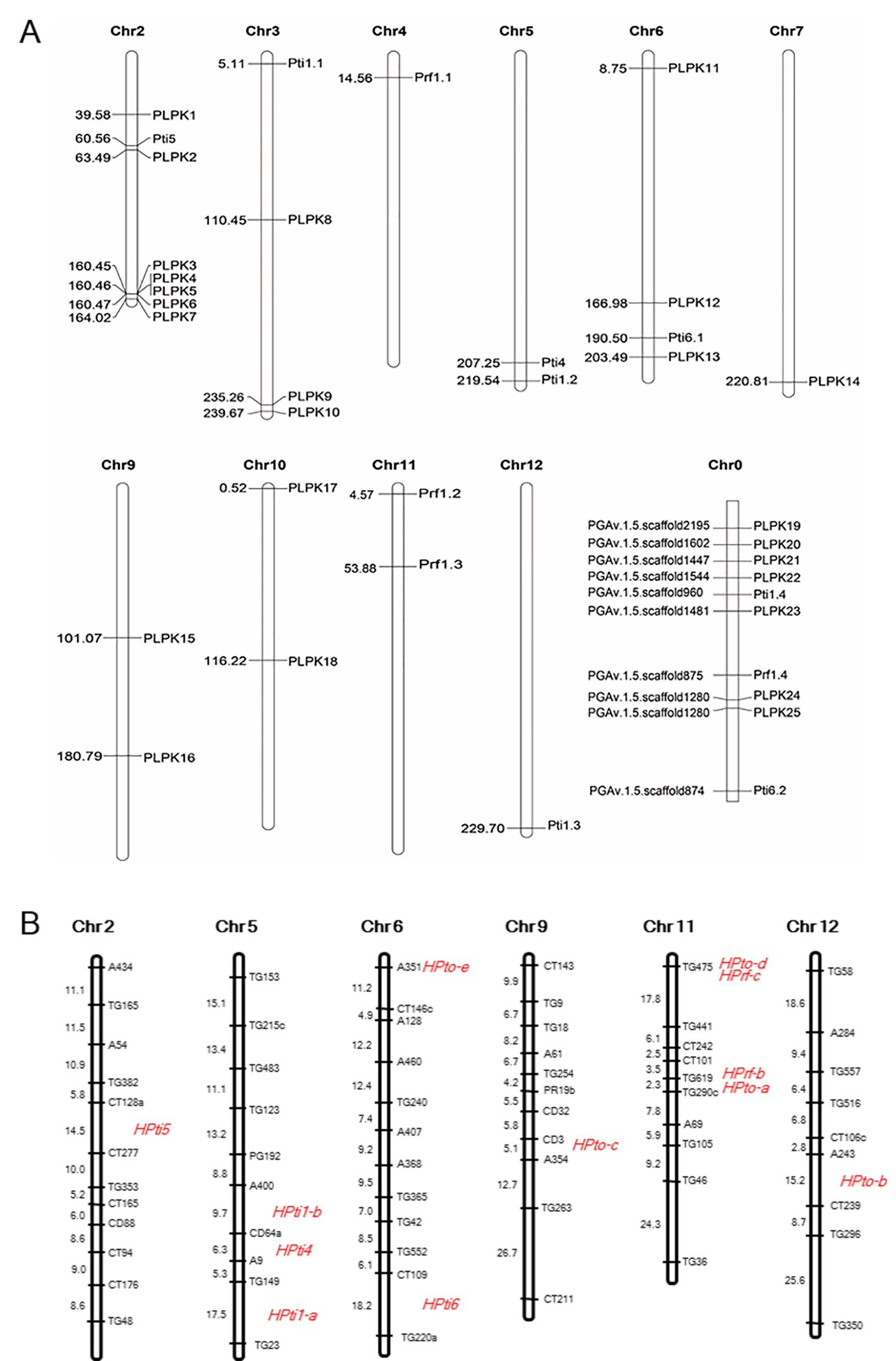

Supplement: S4 Fig — Pepper PLPK gene distribution and linkage map A: The chromosome number is indicated at the top of each chromosome. B: Genetic linkage map containing the Pto pathway genes. Chromosomes 2, 5, 6, 9, 11, and 12 are from the map based on an F2 mapping population from C. annuum and C. chinense [40]. Framework markers are shown on each linkage group in black and the Pto pathway genes are shown in red. (TIF) [file pone.0161545.s004.tif]

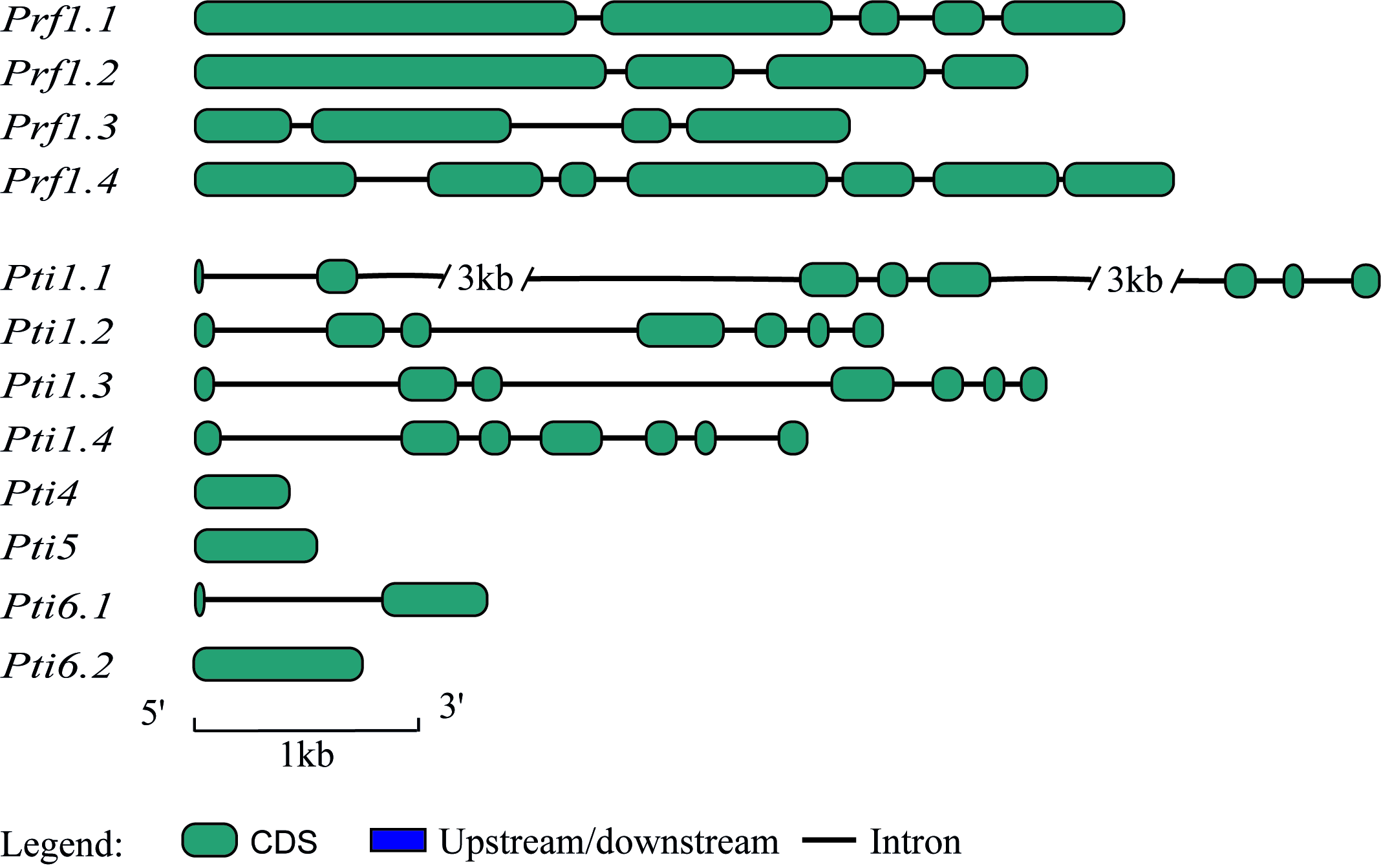

Supplement: S5 Fig — (TIF) [file pone.0161545.s005.tif]

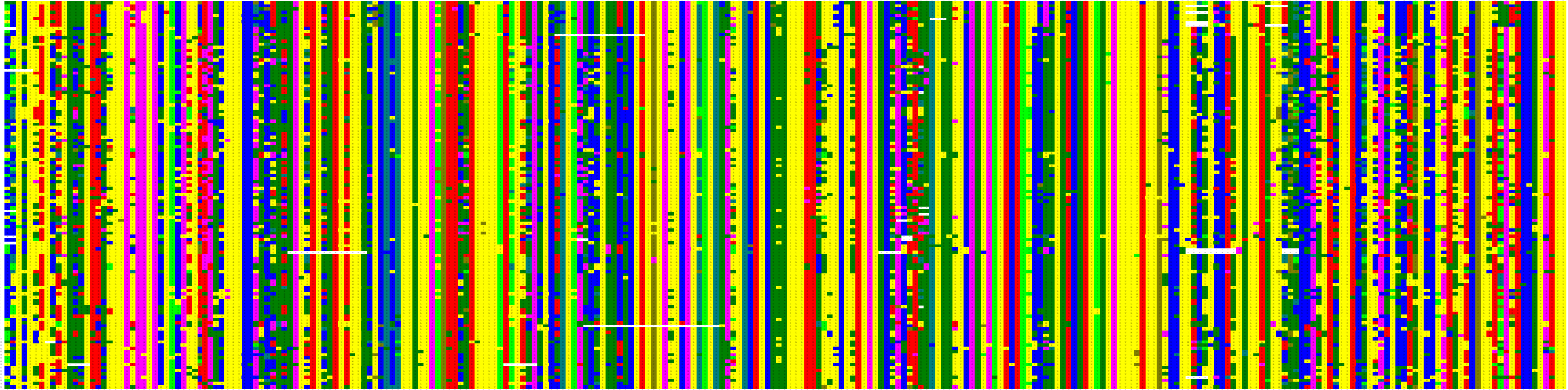

Supplement: S6 Fig — Positive selection analysis was performed by various models as implemented in the Datamonkey webserver (at p value 0.1) and mapped on the alignment.–and n indicates sites under negative and neutral selection pressure, respectively. Sites undergoing episodic selection pressure are indicated with “Ep”. (PDF) [file pone.0161545.s006.pdf]
